# Microbe‐Dependent Exacerbated Alveolar Bone Destruction in Heterozygous Cherubism Mice

**DOI:** 10.1002/jbm4.10352

**Published:** 2020-04-14

**Authors:** Mizuho Kittaka, Tetsuya Yoshimoto, Collin Schlosser, Mikihito Kajiya, Hidemi Kurihara, Ernst J Reichenberger, Yasuyoshi Ueki

**Affiliations:** ^1^ Department of Biomedical Sciences and Comprehensive Care Indiana University School of Dentistry Indianapolis IN USA; ^2^ Indiana Center for Musculoskeletal Health Indiana University School of Medicine Indianapolis IN USA; ^3^ Department of Orthodontics and Dentofacial Orthopedics University of Missouri‐Kansas City, School of Dentistry Kansas City MO USA; ^4^ Department of Periodontal Medicine, Applied Life Sciences Institute of Biomedical & Health Sciences, Graduate School of Biomedical & Health Sciences, Hiroshima University Hiroshima Japan; ^5^ Department of Reconstructive Sciences, School of Dental Medicine University of Connecticut Health Farmington CT USA

**Keywords:** CHERUBISM, ORAL MICROBES, OSTEOCLASTS, PERIODONTITIS, SH3BP2

## Abstract

Cherubism (OMIM#118400) is a craniofacial disorder characterized by destructive jaw expansion. Gain‐of‐function mutations in SH3‐domain binding protein 2 (SH3BP2) are responsible for this rare disorder. We have previously shown that homozygous knock‐in (KI) mice (*Sh3bp2* ^*KI/KI*^) recapitulate human cherubism by developing inflammatory lesions in the jaw. However, it remains unknown why heterozygous KI mice (*Sh3bp2* ^*KI/+*^) do not recapitulate the excessive jawbone destruction in human cherubism, even though all mutations are heterozygous in humans. We hypothesized that *Sh3bp2* ^*KI/+*^ mice need to be challenged for developing exacerbated jawbone destruction and that bacterial stimulation in the oral cavity may be involved in the mechanism. In this study, we applied a ligature‐induced periodontitis model to *Sh3bp2* ^*KI/+*^ mice to induce inflammatory alveolar bone destruction. Ligature placement induced alveolar bone resorption with gingival inflammation. Quantification of alveolar bone volume revealed that *Sh3bp2* ^*KI/+*^ mice developed more severe bone loss (male: 43.0% ± 10.6%, female: 42.6% ± 10.4%) compared with *Sh3bp2* ^*+/+*^ mice (male: 25.8% ± 4.0%, female: 30.9% ± 6.5%). Measurement of bone loss by the cement‐enamel junction–alveolar bone crest distance showed no difference between *Sh3bp2* ^*KI/+*^ and *Sh3bp2* ^*+/+*^ mice. The number of osteoclasts on the alveolar bone surface was higher in male *Sh3bp2* ^*KI/+*^ mice, but not in females, compared with *Sh3bp2* ^*+/+*^ mice. In contrast, inflammatory cytokine levels in gingiva were comparable between *Sh3bp2* ^*KI/+*^ and *Sh3bp2* ^*+/+*^ mice with ligatures. Genetic deletion of the spleen tyrosine kinase in myeloid cells and antibiotic treatment suppressed alveolar bone loss in *Sh3bp2* ^*KI/+*^ mice, suggesting that increased osteoclast differentiation and function mediated by SYK and accumulation of oral bacteria are responsible for the increased alveolar bone loss in *Sh3bp2* ^*KI/+*^ mice with ligature‐induced periodontitis. High amounts of oral bacterial load caused by insufficient oral hygiene could be a trigger for the initiation of jawbone destruction in human cherubism. © 2020 The Authors. *JBMR Plus* published by Wiley Periodicals, Inc. on behalf of American Society for Bone and Mineral Research.

## Introduction

Cherubism (OMIM#118400) is an autosomal‐dominant craniofacial disorder in children characterized by expansive destruction of the maxilla and mandible. Proliferation of fibrous lesions containing large numbers of osteoclasts is responsible for the destruction. Previously, we discovered that heterozygous gain‐of‐function mutations in the signaling adaptor protein SH3‐domain binding protein 2 (SH3BP2) cause cherubism.^1^ We have also shown that homozygous knock‐in (KI) mice (*Sh3bp2* ^*KI/KI*^) harboring the most common mutation in cherubism patients recapitulate the many features of human cherubism by developing spontaneous fibrous inflammatory lesions and by exhibiting increased osteoclast formation in the jaw.^2^ Homozygous mutant macrophages exhibit hyper‐responsiveness against bacterial pathogens via Toll‐like receptors (TLRs), and heterozygous and homozygous cherubism mutations promote osteoclastogenesis induced by RANKL and TNF‐ɑ.^2^, ^3^, ^4^ The mode of inheritance of cherubism lesion development is different between humans and mice. In humans, cherubism lesions develop as an autosomal dominant trait, whereas heterozygosity of the SH3BP2 gain‐of‐function mutation is not sufficient to develop spontaneous cherubism lesions in our mouse model.^2^ As a result, most studies in the cherubism mouse model have been conducted in homozygous *Sh3bp2* ^*KI/KI*^ mice, where they express stronger phenotypes than human cherubism.

Currently, it remains unknown why heterozygous KI mice (*Sh3bp2* ^*KI/+*^) do not recapitulate the destructive jawbone phenotype in human cherubism. In this study, we hypothesized that a bacterial challenge might be required for *Sh3bp2* ^*KI/+*^ mice to develop exacerbated jawbone destruction. We induced ligature‐induced periodontitis in *Sh3bp2* ^*KI/+*^ mice to cause inflammatory jawbone destruction. Ligature placement induced more severe alveolar bone resorption in *Sh3bp2* ^*KI/+*^ mice than in WT mice. In male *Sh3bp2* ^*KI/+*^ mice, but not in females, the number of TRAP‐positive osteoclasts on the affected alveolar bone surface (BS) was higher than in WT mice. However, inflammatory cytokine levels in the gingiva were comparable between *Sh3bp2* ^*KI/+*^ and WT mice with ligatures. We also show that the increased alveolar bone loss in *Sh3bp2* ^*KI/+*^ mice is spleen tyrosine kinase‐ (SYK‐) dependent and improves when treated with antibiotics. These data suggest that increased osteoclast formation and function is responsible for exacerbated alveolar bone loss in male *Sh3bp2* ^*KI/+*^ mice and that the increased bone resorption capability of osteoclasts is sufficient for increased alveolar bone loss in female *Sh3bp2* ^*KI/+*^ mice. Oral microbe‐dependent alveolar bone loss in *Sh3bp2* ^*KI/+*^ mice suggests that a high oral bacterial load, for example, caused by insufficient oral hygiene, could be a trigger of jawbone destruction in human cherubism and that reduction of bacterial load by increased oral hygiene could reduce the risk of severe jawbone destruction in cherubism patients.

## Materials and Methods

### Mice

All animal experiments were conducted under animal protocols approved by the IACUCs of Indiana University and the University of Missouri‐Kansas City. *Sh3bp2* ^*KI/+*^ mice in a C57BL/6J background were reported previously.^2^ *Syk* ^*fl/fl*^ mice (017309) and *LysM‐Cre* mice (004781) were obtained from the Jackson Laboratory (Bar Harbor, ME, USA). All mice were bred and housed under specific‐pathogen‐free conditions.

### A ligature‐induced periodontitis model^5^

The maxillary left second molar of 10‐week‐old mice was ligatured with 5‐0 silk suture (Ethicon, Somerville, NJ, USA) for 5 days. The right second molar was unligated for the control of alveolar bone volume analysis. For the suppression of oral microbes, mice were treated with an antibiotic cocktail in their drinking water (1.0 g/L ampicillin, 0.5 g/L vancomycin, 1.0 g/L kanamycin, 1.0 g/L metronidazole) *ad libitum* from 5 days before to 5 days after ligature placement.

### Bacterial colony formation assay

Ligatures were recovered 5 days after ligature placement. Accumulated bacteria on ligatures were suspended in 500 μL of sterile PBS by vortexing for 3 min. Bacterial suspensions were serially diluted and cultured on trypticase soy agar supplemented with 5% sheep blood (BD Bioscience, San Jose, CA, USA) at 37°C for 48 hours in aerobic or anaerobic conditions. Anaerobic conditions were created by using the AnaeroPack system (Mitsubishi Gas Chemical, Tokyo, Japan). The number of CFUs was normalized by the length of the ligatures.

### μCT analysis^5^

Maxillas were fixed with 4% paraformaldehyde (PFA) for 24 hours and soaked in 70% ethanol before scanning with the Skyscan1174 (Bruker, Kontich, Belgium). The following conditions were used: 80 kV, 6.67‐μm pixel size, and 0.4‐degree rotation step, and 3000‐ms exposure time. Scanned data were reconstructed with NRecon software (Bruker, Kontich, Belgium) in the dynamic range from 0 to 0.22. The 3D images were aligned with the DataViewer (Bruker, Kontich, Belgium). Alveolar bone between two buccal roots underneath the maxillary second molar, which is composed of 21 slices (approximately 140‐μm thickness), was segmented, and the bone volume (BV) was measured by CT‐Analyzer (Bruker, Kontich, Belgium) with a threshold value of 44 (Supplemental Fig. S9). The following formula: [(BV of unligated side − BV of ligated side)/BV of unligated side] × 100, was used to calculate the percentage of reduction rate (susceptibility for alveolar bone loss).

### Taxonomic classification of oral microbe^5^

Five days after ligature placement, each ligature was recovered and vortexed in sterile PBS. Bacterial DNA was isolated using the Meta‐G‐Nome DNA Isolation Kit (Epicentre, Madison, WI, USA) and used for 16S rDNA analysis (BGI America, Cambridge, MA, USA). The V4 region of 16S rDNA was amplified by PCR for next‐generation sequencing library construction. Taxonomic classification was performed by Ribosomal Database Project Classifier v. 2.2.^6^

### RNA isolation^5^

For RNA isolation by RiboZol reagent (Amresco, Solon, OH, USA), 1‐mm × 3‐mm palatal gingival tissue and alveolar bone tissue surrounding the maxillary three molars of ligated mice were used separately. RNA samples from unligated mice were used for controls. Gingival tissues were homogenized by a tissue grinder. After removing soft tissues, jawbones were snap‐frozen in liquid nitrogen and crushed into powder using a tissue pulverizer (Cellcrusher Limited, Portland, OR, USA).

### qRT‐PCR analysis^5^

There were 500 ng of total RNA used for cDNA synthesis (High Capacity cDNA Reverse Transcription Kit; Applied Biosystems, Foster City, CA, USA). Maxima SYBR Green qPCR Master Mix (Thermo Fisher Scientific, Waltham, MA, USA) was used for qPCR reactions in the StepOnePlus Real‐Time PCR System (Applied Biosystems). Primer sequences are listed in Supplemental Table S1. Relative gene expression levels were calculated using the standard curb method. *Hprt* expression level was used for normalization of target gene expression.

### Histology^5^

Maxillas were decalcified with EDTA (0.5M, pH 7.2) after fixation with 4% PFA for 24 hours and embedded in paraffin. Sections cut in the sagittal plane were subjected to H&E and tartrate‐resistant acid phosphatase (TRAP) staining.

### Bone histomorphometry^5^

Alveolar bone tissues between two buccal roots of the maxillary second molar were stained for TRAP. The number of osteoclasts (N.Oc), the osteoclast surface (Oc.S), and the BS were measured using Bioquant Osteo software (Bioquant Image Analysis Corp, Nashville, TN, USA). Bone tissues from unligated mice or the unligated right side were used as controls. In each mouse, results from two sections separated by 20 to 50 μm were averaged. Measurements were performed by personnel blinded to genotypes.

### Osteoclast differentiation assay^5^

Bone marrow cells in the tibia, femur, and ilium were collected from 7‐ to 8‐week‐old mice. Red blood cell‐free bone marrow cells were incubated with α‐MEM containing 10% FBS and penicillin/streptomycin for 3 hours on Petri dishes. Nonadherent bone marrow cells were collected and cultured on Petri dishes with M‐CSF (25 ng/mL; PeproTech, Rocky Hill, NJ, USA) for 3 days to grow bone marrow‐derived M‐CSF‐dependent macrophages (BMMs). BMM cells were harvested and cultured on 48‐well plates (2.5 × 10^4^ cells/well) and further stimulated with M‐CSF (25 ng/mL) and RANKL (50 ng/mL; PeproTech) for 3 days.

### Osteoclast resorption assay^5^

BMMs were seeded on Osteo assay plates (Corning, Corning, NY, USA) or dentin slices in 96‐well plates at a density of 8.3 × 10^3^ cells/well, and further stimulated with M‐CSF (25 ng/mL) and RANKL (50 ng/mL) for 7 days. After removing cells with 10% bleach, nonresorbed areas were identified by von Kossa staining. Dentin slices were stained with toluidine blue to visualize resorption pits after removing cells by scrubbing with a toothbrush and using a Kimwipe. For quantitating the depth of the resorption pits, decalcified dentin slices with EDTA (0.5M pH = 7.2) were embedded in paraffin and stained with H&E after sectioning. Four sections separated by at least 100 μm were used to measure depth by ImageJ software (NIH, Bethesda, MD, USA; https://imagej.nih.gov/ij/).

### Statistics

The two‐tailed unpaired Student's *t* test and Mann–Whitney *U* test were used to compare two groups. One‐way ANOVA with Tukey–Kramer post hoc test was used to compare three or more groups. GraphPad Prism (ver. 5; GraphPad Software, La Jolla, CA, USA) and SPSS (ver. 20; IBM, Armonk, NY, USA) were used for all statistical analyses. The *p* values <0.05 were considered significant.

## Results

### Heterozygous cherubism mutation increases susceptibility to alveolar bone loss in ligature‐induced periodontitis

**Figure 1:**
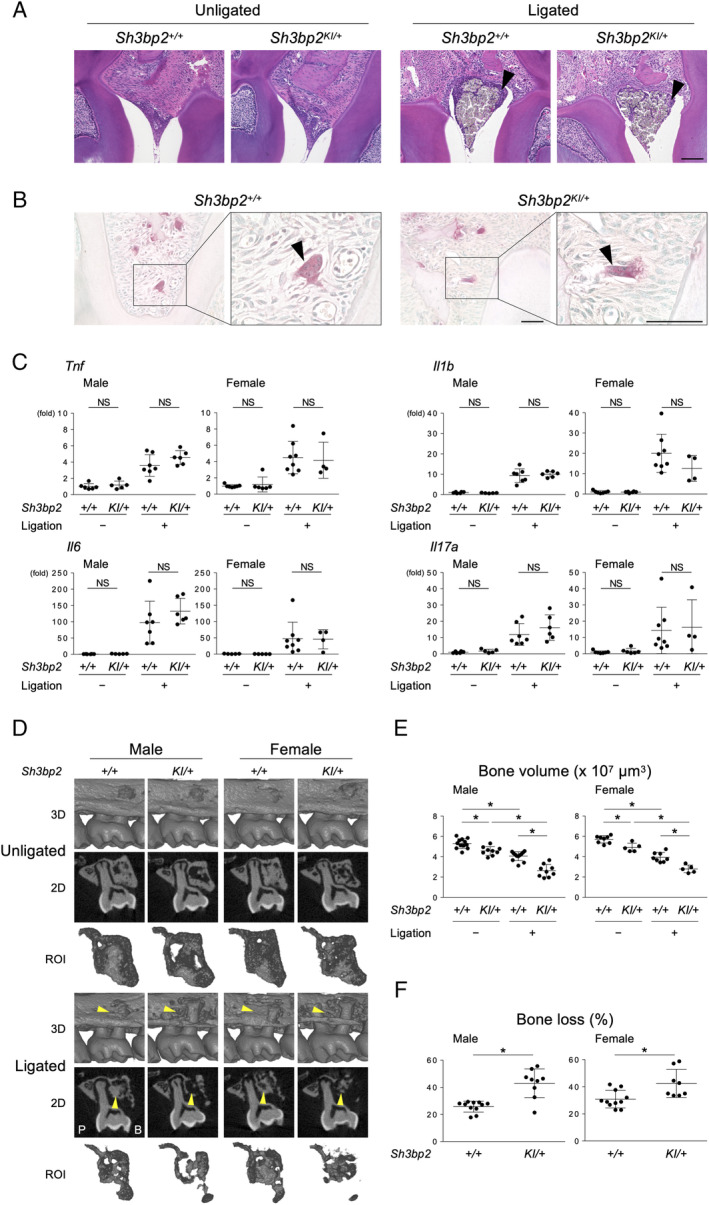
The gain‐of‐function mutation of SH3BP2 exacerbates alveolar bone loss in ligature‐induced periodontitis. (*A*) H&E staining of gingival tissue in males between the first and second molars. Scale bar = 100 μm. Arrowheads indicate inflammatory infiltrates surrounding the ligated silk sutures. (*B*) Tartrate‐resistant acid phosphatase (TRAP) staining of tissues in males between two buccal roots. Arrowheads indicate multinucleated cells that are not located on the alveolar bone surface. Scale bar = 50 μm. (*C*) qPCR analysis with RNA isolated from the gingiva. Average levels in unligated WT mice were set as 1. (*D*) 3D μCT images surrounding the maxillary second molar (top), 2D coronal plane μCT images in the middle of the maxillary second molar (middle), and the region of interest (ROI) used for bone volume measurement (bottom). Arrowheads indicate the areas of alveolar bone loss. P = Palatal side; B = buccal side. (*E*) Alveolar bone volume between two buccal roots underneath the maxillary second molar. (*F*) Percentage of bone loss against the contralateral unligated side. ANOVA with Tukey–Kramer post hoc test. Mean ± SD. *p < 0.05. NS = not significant.

To investigate whether bacterial challenge exacerbates alveolar bone loss in heterozygous cherubism mice (*Sh3bp2* ^*KI/+*^), we used ligature‐induced periodontitis. Ligature placement caused comparable levels of gingival tissue inflammation with the accumulation of inflammatory cells surrounding ligatures in WT (*Sh3bp2* ^*+/+*^) and *Sh3bp2* ^*KI/+*^ mice (Fig. 1 *A*). A few TRAP‐positive multinucleated cells were observed within connective tissues underneath the ligated second molar in *Sh3bp2* ^*+/+*^ and *Sh3bp2* ^*KI/+*^ mice (Fig. 1 *B*). qPCR analysis of gingival tissue showed that expression levels of *Adgre* and *Itgam* (macrophage markers), *Ly6g* (a neutrophil marker), *Cxcr2* (a chemokine receptor expressed in neutrophils), *Cxcr4* (a chemokine receptor expressed in leukocytes), and *Cd3g* (a T‐cell marker) were not significantly changed between ligated *Sh3bp2* ^*+/+*^ and *Sh3bp2* ^*KI/+*^ mice (Supplemental Fig. S1). Ligatured mice showed increased levels of inflammatory cytokines in gingival tissues. However, heterozygous cherubism mice did not show increased expression levels compared with WT mice (Fig. 1 *C*).

We performed μCT analysis of the maxilla and found that ligature placement caused more severe alveolar bone erosion in *Sh3bp2* ^*KI/+*^ mice compared with *Sh3bp2* ^*+/+*^ mice (Fig. 1 *D*). The basal level of alveolar bone volume without ligature was lower in *Sh3bp2* ^*KI/+*^ mice (Fig. 1 *E*; unligated), which is consistent with our previous report that *Sh3bp2* ^*KI/+*^ mice exhibit osteopenia in long bones because of increased osteoclast formation.^2^ Induction of periodontitis further decreased the alveolar bone volume in *Sh3bp2* ^*KI/+*^ and *Sh3bp2* ^*+/+*^ mice (Fig. 1 *E*; ligated) and the percentage of alveolar bone loss in the ligated side compared with the unligated side (susceptibility to bone loss) was higher in *Sh3bp2* ^*KI/+*^ mice (Fig. 1 *F*). The increase in the cement‐enamel junction–alveolar bone crest distance by ligatures was comparable between *Sh3bp2* ^*KI/+*^ and *Sh3bp2* ^*+/+*^ mice (Supplemental Fig. S2).

Next, we examined whether the gain‐of‐function of SH3BP2 causes dysbiosis in the oral microbiome. We determined the taxonomic composition of bacteria accumulated in the silk suture. Taxonomic analysis showed that the bacterial flora was mostly composed of *Pasteurellales*, *Lactobacillales*, *Bacteroidales*, *Bifidobacteriales*, and *Erysipelotrichales*, and the composition of bacteria was not significantly changed between *Sh3bp2* ^*KI/+*^ and *Sh3bp2* ^*+/+*^ mice (Supplemental Fig. S3). This result suggests that *Sh3bp2* ^*KI/+*^ mutation does not change the composition of the oral microbiome and that a dysbiotic change is not a cause for the increased alveolar bone loss in *Sh3bp2* ^*KI/+*^ mice.

### Ligature‐induced periodontitis increases osteoclast formation in male mice with heterozygous cherubism mutation

**Figure 2:**
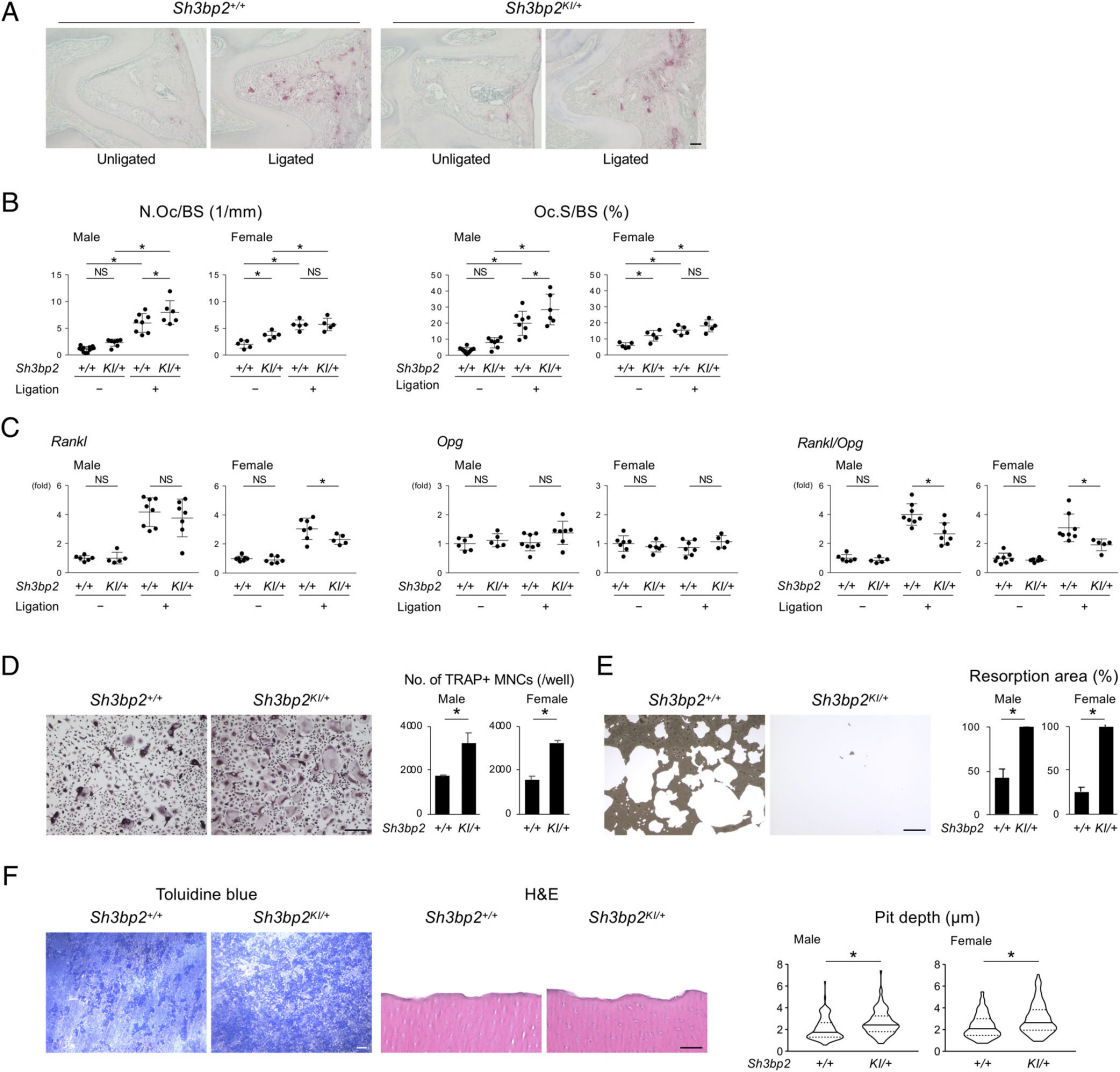
Gain‐of‐function of SH3BP2 increases differentiation and bone‐resorbing capacity of osteoclasts. (*A*) Tartrate‐resistant acid phosphatase (TRAP) staining of alveolar bone in males between two buccal roots of the ligated second molar. Scale bar = 100 μm. (*B*) Histomorphometric analysis for TRAP‐positive cells on the alveolar bone surface. (*C*) qPCR analysis for *Rankl* and *Opg* expression and their ratio. RNA was isolated from the alveolar bone. Average levels in unligated WT mice were set as 1. (*D*) TRAP staining of male bone marrow‐derived M‐CSF‐dependent macrophages (BMMs) stimulated with 50 ng/mL RANKL for 5 days and quantitation of the TRAP‐positive multinucleated cells (MNCs) per well. *n* = 4. Scale bar = 100 μm. (*E*) Resorption assay for calcium phosphate. BMMs from male mice stimulated with 50 ng/mL RANKL were cultured for 7 days. Cells were removed, and remaining calcium phosphate was stained with silver nitrate to visualize the nonresorbed area. Resorbed areas were measured by ImageJ software (NIH, Bethesda, MD, USA; https://imagej.nih.gov/ij/). *n* = 4. Scale bar = 100 μm. (*F*) Resorption assay using dentin slices. BMMs from male mice stimulated with RANKL were cultured for 7 days. Cells were removed, and dentin slices were stained with toluidine blue for observation by stereomicroscope. Dentine slices were further decalcified and sectioned for H&E staining. Pit depth was measured with cross‐section H&E images of the dentin slice using ImageJ software. *n* = 100 in males and 100 in females. Scale bar = 100 μm (toluidine blue staining) and 10 μm (H&E staining). Mean ± SD. *p < 0.05. NS = not significant. ANOVA with Tukey–Kramer post hoc test for (*B,C*), Student's *t* test for (*D,E*), Mann–Whitney *U* test for (*F*).

Ligature placement induced osteoclast formation on the alveolar BS underneath the maxillary second molar in both *Sh3bp2* ^*KI/+*^ and *Sh3bp2* ^*+/+*^ mice (Fig. 2 *A*). Histomorphometric analysis showed that the N.Oc/BS and Oc.S/BS are higher in male *Sh3bp2* ^*KI/+*^ mice, but not in female mice, compared with *Sh3bp2* ^*+/+*^ mice (Fig. 2 *B*). However, the *Rankl* and *Osteoprotegerin* (*Opg*) ratio in the jawbone of male and female *Sh3bp2* ^*KI/+*^ mice was even lower than in *Sh3bp2* ^*+/+*^ mice (Fig. 2 *C*). Expression levels of other osteoclast‐associated genes were not significantly changed in male and female *Sh3bp2* ^*KI/+*^ mice with ligatures compared with *Sh3bp2* ^*+/+*^ mice with ligatures (Supplemental Fig. S4). Consistent with our previous results,^2^ RANKL‐stimulated *Sh3bp2* ^*KI/+*^ BMMs differentiated more efficiently into TRAP‐positive multinucleated osteoclasts than *Sh3bp2* ^*+/+*^ BMMs (Fig. 2 *D*). *Sh3bp2* ^*KI/+*^ osteoclasts had a higher mineral absorption capacity than *Sh3bp2* ^*+/+*^ osteoclasts (Fig. 2 *E*), and deeper pits were created by *Sh3bp2* ^*KI/+*^ osteoclasts when compared with *Sh3bp2* ^*+/+*^ osteoclasts (Fig. 2 *F*). These data indicate that increased osteoclast formation and function is responsible for increased alveolar bone loss in male *Sh3bp2* ^*KI/+*^ mice with ligature‐induced periodontitis, whereas increased osteoclast function is sufficient to cause a more significant alveolar bone loss in female *Sh3bp2* ^*KI/+*^ mice.

### SYK deletion in myeloid cells prevents increased alveolar bone loss in heterozygous cherubism mice with ligature‐induced periodontitis

**Figure 3:**
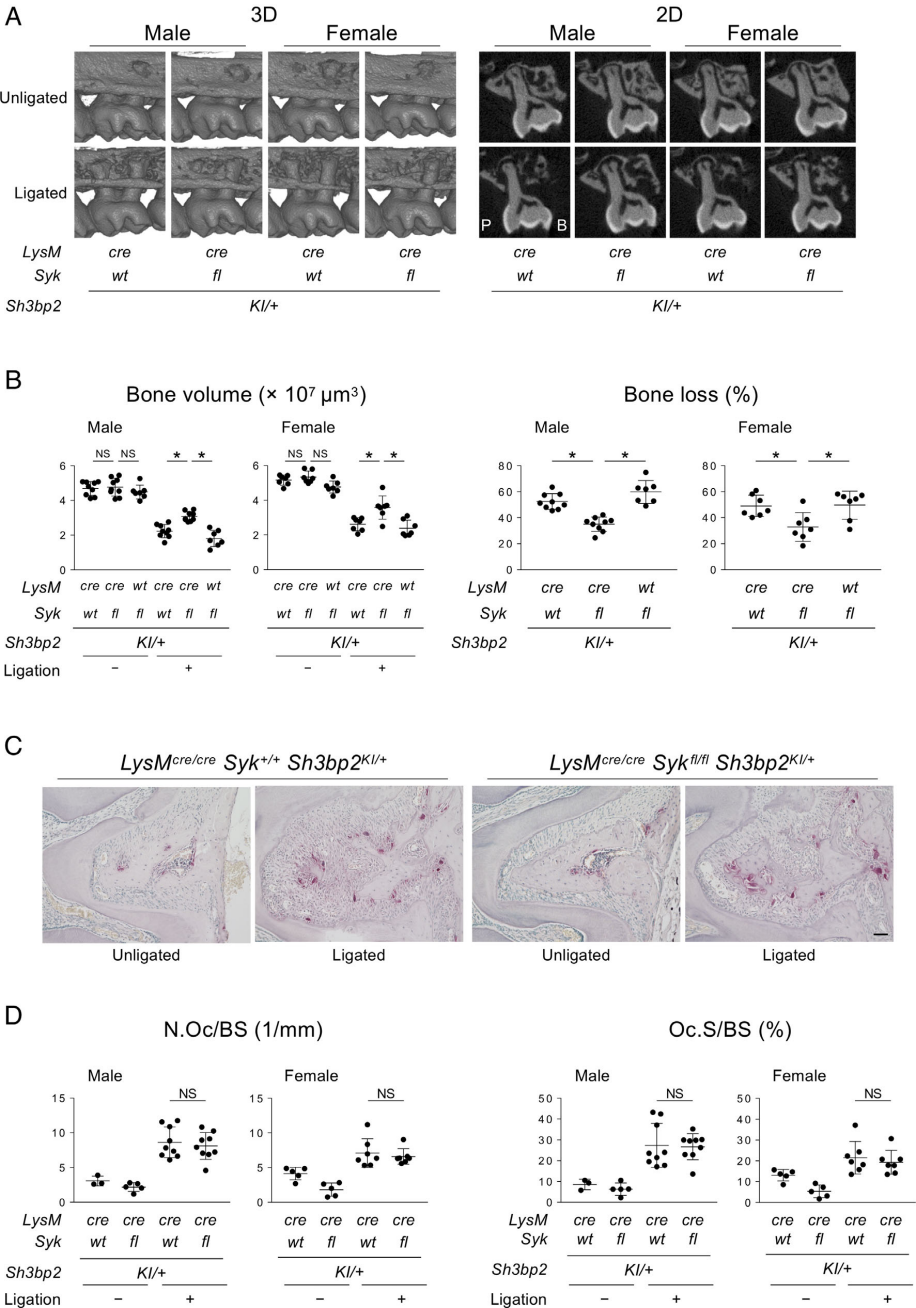
Spleen tyrosine kinase (SYK) deletion in myeloid cells decreases susceptibility to bone loss in *Sh3bp2* ^*KI/+*^ mice with ligature‐induced periodontitis. (*A*) 3D μCT images surrounding the maxillary second molar (left) and 2D coronal plane μCT images in the middle of the maxillary second molar (right). (*B*) Alveolar bone volume and percentage of bone loss against the contralateral unligated side. (*C*) TRAP staining of alveolar bone in males between two buccal roots of the ligated second molar. Scale bar = 100 μm. (*D*) Histomorphometric analysis for TRAP‐positive cells. ANOVA with Tukey–Kramer post hoc test. Mean ± SD. *p < 0.05. NS = not significant; *+ = +/+*; *cre = cre/cre; fl = fl/fl*.

SYK plays a crucial role in the mechanism for enhanced osteoclast differentiation by the gain‐of‐function mutation in SH3BP2.^2^, ^4^ We have also shown that a lack of SYK in *LysM‐Cre‐*expressing cells rescues homozygous cherubism mice from inflammatory bone destruction.^3^ These results led us to investigate whether SYK is required for SH3BP2 gain‐of‐function to increase osteoclast function in periodontitis. To examine whether SYK in myeloid cells mediates the increase of susceptibility to alveolar bone loss in *Sh3bp2* ^*KI/+*^ mice, we depleted SYK in *LysM‐Cre‐*expressing cells in *Sh3bp2* ^*KI/+*^ mice (*LysM‐Cre Syk* ^*fl/fl*^ *Sh3bp2* ^*KI/+*^). SYK deletion in *LysM‐Cre‐*expressing cells suppressed alveolar bone loss (Fig. 3 *A*) and reduced the increased susceptibility of alveolar bone loss in *Sh3bp2* ^*KI/+*^ mice to the level of *Sh3bp2* ^*+/+*^ mice (Figs. 1 *F* and 3 *B*). TRAP staining showed that osteoclasts form in the absence of SYK in myeloid cells (Fig. 3 *C*). Histomorphometric analysis showed that osteoclast parameters in *Sh3bp2* ^*KI/+*^ mice are comparable to *Sh3bp2* ^*+/+*^ mice (Fig. 3 *D*). Expression levels of osteoclast‐associated genes were not significantly changed in male and female *LysM‐Cre Syk* ^*fl/fl*^ *Sh3bp2* ^*KI/+*^ mice with ligatures compared with *LysM‐Cre Syk* ^*+/+*^ *Sh3bp2* ^*KI/+*^ and *Syk* ^*fl/fl*^ *Sh3bp2* ^*KI/+*^ mice with ligatures except for *Rankl* in females and *Acp5* (Supplemental Fig. S5). These results suggest that the SH3BP2^KI/+^–SYK signaling axis contributes to the increased bone‐resorbing capacity of osteoclasts and that SYK deletion reduces alveolar bone resorption in *Sh3bp2* ^*KI/+*^ mice by suppressing osteoclast function, not numbers.

### Increased alveolar bone loss in heterozygous cherubism mice with ligature‐induced periodontitis is microbe‐dependent

**Figure 4:**
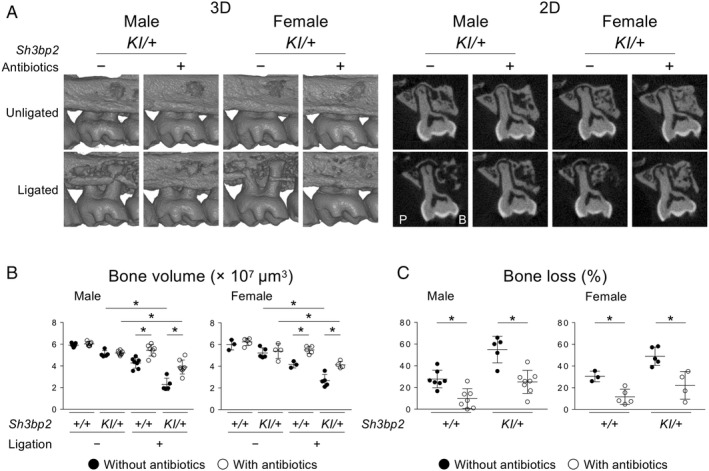
Antibiotic treatment suppresses alveolar bone loss in ligature‐induced periodontitis both in WT and *Sh3bp2* ^*KI/+*^ mice. (*A*) 3D μCT images surrounding the maxillary second molar (left) and 2D coronal plane μCT images in the middle of the maxillary second molar (right). P = Palatal side; B = buccal side. (*B*) Alveolar bone volume between two buccal roots underneath the maxillary second molar. (*C*) Percentage of bone loss against the contralateral unligated side. ANOVA with Tukey–Kramer post hoc test. Mean ± SD. *p < 0.05. NS = not significant.

We hypothesized that the bacterial load surrounding ligatures is responsible for increased alveolar bone destruction in *Sh3bp2* ^*KI/+*^ mice and that a decrease in bacterial load can suppress bone destruction. Bacterial load was successfully decreased by administering antibiotics via drinking water (Supplemental Fig. S6). We discovered that antibiotic treatment suppresses alveolar bone resorption on the ligated side (Fig. 4 *A,B*) and decreases the susceptibility to bone loss in *Sh3bp2* ^*KI/+*^ and *Sh3bp2* ^*+/+*^ mice (Fig. 4 *C*). Antibiotics also reduced expression levels of *Tnf*, *Il1b*, *Il6*, and *Il17a* in gingival tissue of *Sh3bp2* ^*KI/+*^ and *Sh3bp2* ^*+/+*^ mice in a gender‐dependent manner (Supplemental Fig. S7). Together, the data show that increased bacterial load surrounding ligatures is a critical trigger for increased alveolar bone loss in *Sh3bp2* ^*KI/+*^ mice with ligature‐induced periodontitis.

## Discussion

Cherubism is a genetic disorder characterized by jawbone destruction by fibro‐osseous lesions containing large numbers of proliferating fibrous stromal cells and multinucleated osteoclasts. Previously, we have shown that heterozygous mutations in the adaptor protein SH3BP2 are responsible for cherubism.^1^ In the cherubism mouse model, the homozygous mutation is required to exhibit spontaneous jawbone destruction by inflammation.^2^ However, homozygous mice develop a phenotype that extends beyond the craniofacial skeleton, which is much more severe than human cherubism and has not been observed in human cherubism patients. In contrast, heterozygous cherubism mice do not exhibit jawbone destruction, but they do exhibit systemic osteopenia caused by increased osteoclast activation. As a result, the reason why heterozygous cherubism mutant mice do not recapitulate the human cherubism phenotype could not be well‐explained.

We and others have shown that macrophages with cherubism mutations increase responsiveness to microbial pathogens via TLRs.^3^, ^7^ The results suggested that the bacterial load in the oral cavity may contribute to triggering jawbone destruction in human cherubism. To investigate whether the bacterial load is a significant trigger for heterozygous cherubism mice to develop exacerbated jawbone destruction, we applied ligature‐induced periodontitis to heterozygous *Sh3bp2* ^*KI/+*^ mice. We found that this challenge caused more severe alveolar destruction in *Sh3bp2* ^*KI/+*^ mice compared with *Sh3bp2* ^*+/+*^ mice, which is reminiscent of jawbone destruction in human cherubism. However, there was no difference in inflammatory cytokine levels in gingival tissue between *Sh3bp2* ^*KI/+*^ and *Sh3bp2* ^*+/+*^ mice treated with ligatures. Also, only male *Sh3bp2* ^*KI/+*^ mice showed increased osteoclast induction after ligature placement. Because the heterozygous cherubism mutation renders osteoclast progenitors with increased capability for osteoclast differentiation and bone resorption in vitro, the data suggest that the heterozygous mutation promotes alveolar bone resorption by increasing osteoclast differentiation and function in male *Sh3bp2* ^*KI/+*^ mice and by increasing osteoclast function rather than osteoclast differentiation and number in female *Sh3bp2* ^*KI/+*^ mice. Indeed, pits created by male and female *Sh3bp2* ^*KI/+*^ osteoclasts are deeper than *Sh3bp2* ^*+/+*^ osteoclasts, suggesting that *Sh3bp2* ^*KI/+*^ osteoclasts have higher bone‐resorption capacity than *Sh3bp2* ^*+/+*^ osteoclasts. Further studies are required to investigate the sex‐dependent impact of the *Sh3bp2* ^*KI/+*^ mutation on osteoclast formation in ligature‐induced periodontitis and to find evidence supporting increased osteoclast function in vivo.

We showed that the genetic deletion of SYK in *LysM‐Cre‐*expressing cells suppresses alveolar bone resorption in *Sh3bp2* ^*KI/+*^ mice with ligature‐induced periodontitis. The result suggests that SYK is critically involved in the exacerbation of alveolar bone resorption in *Sh3bp2* ^*KI/+*^ mice, and provides another piece of evidence that SYK is a critical downstream kinase of SH3BP2 in myeloid cells.^3^ We administered a novel SYK inhibitor GS‐9973^8^ intraperitoneally starting from 1 day before periodontitis induction. We found that 200 mg/kg GS‐9973 reduces alveolar bone loss in female *Sh3bp2* ^*KI/+*^ mice, but not in males, with periodontitis (Supplemental Fig. S8), suggesting that SYK activation is more critical in female *Sh3bp2* ^*KI/+*^ osteoclasts to regulate bone resorption in ligature‐induced periodontitis. Combined administration of SYK and pro‐osteoclastogenic cytokine inhibitors may be required to suppress alveolar bone loss in male *Sh3bp2* ^*KI/+*^ mice effectively. The sex difference in the in vivo susceptibility to the SYK inhibitor needs to be further investigated.

Because antibiotic treatment suppresses alveolar bone resorption in *Sh3bp2* ^*KI/+*^ mice, the accumulation of oral bacteria is a primary cause of exacerbated alveolar bone resorption in *Sh3bp2* ^*KI/+*^ mice. Oral pathogenic bacteria such as *Porphyromonas gingivalis* accumulate in the gingival sulcus and cause harmful immune responses via the junctional epithelium, resulting in alveolar bone loss.^9^, ^10^ Therefore, the formation of a sulcus after tooth eruption, which provides the niche for oral bacteria to grow, may explain why the development of jawbone destruction in young cherubism patients typically starts after tooth eruption. Further studies are needed to determine the pathological association between periodontitis and fibrous lesion development in the cherubism mouse model and human cherubism.

In summary, our data suggest that a high oral bacterial load caused by, for example, insufficient oral hygiene or severe periodontal diseases, can be a trigger for the development of jaw‐dominant bone destruction in human cherubism. In other words, cherubism patients who already have lesions or children who were diagnosed to have a cherubism mutation in *SH3BP2*, but have not yet developed lesions, may benefit from intensive oral care, such as frequent tooth brushing and antibacterial mouthwash, or regular debridement of oral bacterial plaques.

## Disclosures

The authors have declared that no conflict of interest exists.

## Supporting information


**Table S1**. Primer list for SYBR Green qPCR

**Figure S1**. qPCR analysis of immune cell marker genes with RNA isolated from gingival tissue. Data are presented as mean ± SD. *p < 0.05. NS = not significant. ANOVA with Tukey‐Kramer post hoc test. NS = not significant.

**Figure S2**. The distance between cementoenamel junction and alveolar bone crest (CEJ‐ABC) of the second molar was measured on the coronal plane 2D μCT image. Data are shown as the total distance of 4 measurement points (medial buccal, medial palatal, distal buccal, and distal palatal). Bone loss (μm) was calculated by subtracting the distance on the unligated side from the ligated side. Student's *t*‐test. NS = not significant.

**Figure S3**. Taxonomic analysis of bacterial composition in ligatures on an order level. Silk sutures placed to induce periodontitis were recovered after 5 days and bacterial DNA was isolated. The V4 region of 16S rDNA was amplified by PCR for library construction and followed by sequencing analysis. Graphs show taxonomic bacterial composition on an order level. Average numbers from 3 independent samples (n = 3) were used for the graphs. *p* values were calculated with Student's *t*‐test.

**Figure S4**. qPCR analysis of osteoclast‐associated genes with RNA isolated from alveolar bone. Data are presented as mean ± SD. *p < 0.05. NS = not significant. ANOVA with Tukey‐Kramer post hoc test. NS = not significant.

**Figure S5**. qPCR analysis of osteoclast‐associated genes with RNA isolated from alveolar bone. Data are presented as mean ± SD. *p < 0.05. NS = not significant. ANOVA with Tukey‐Kramer post hoc test. NS = not significant.

**Figure S6**. Decreased bacterial load by antibiotics treatment. (A) Bacterial colony formation assay. Wild‐type mice were treated with or without antibiotics in drinking water starting at 5 days before ligature placement, and 1/9000 of the bacteria on ligatures were cultured on blood agar plates under aerobic and anaerobic conditions. (B) The number of colonies per 1 mm of ligatures. CFU: colony‐forming unit. Data are presented as mean ± SD. *p < 0.05. Student's *t*‐test.

**Figure S7**. qPCR analysis of inflammatory cytokine genes with RNA isolated from gingival tissue. Data are presented as mean ± SD. *p < 0.05. NS = not significant. ANOVA with Tukey‐Kramer post hoc test. NS = not significant.

**Figure S8**. Effect of SYK inhibitor GS‐9973 in alveolar bone. (A) MicroCT images of alveolar bone underneath the maxillary second molar. (B) Alveolar bone volume underneath the maxillary second molar. (C) Percentage (%) of bone loss against contralateral unligated side. Data are presented as mean ± SD. *p < 0.05. NS = not significant. ANOVA with Tukey‐Kramer post hoc test. NS = not significant.

**Figure S9**. Schematic images of the area that was used for the assessment of bone volume. Alveolar bone of the maxillary second molar between mesiobuccal root and distobuccal root was measured by microCT.
