# Supplementary figures and images for: Microbe‐Dependent Exacerbated Alveolar Bone Destruction in Heterozygous Cherubism Mice
Source: JBMR Plus. 2020 Apr 14;4(6):e10352. doi: 10.1002/jbm4.10352 (PMC7285758; doi:10.1002/jbm4.10352)

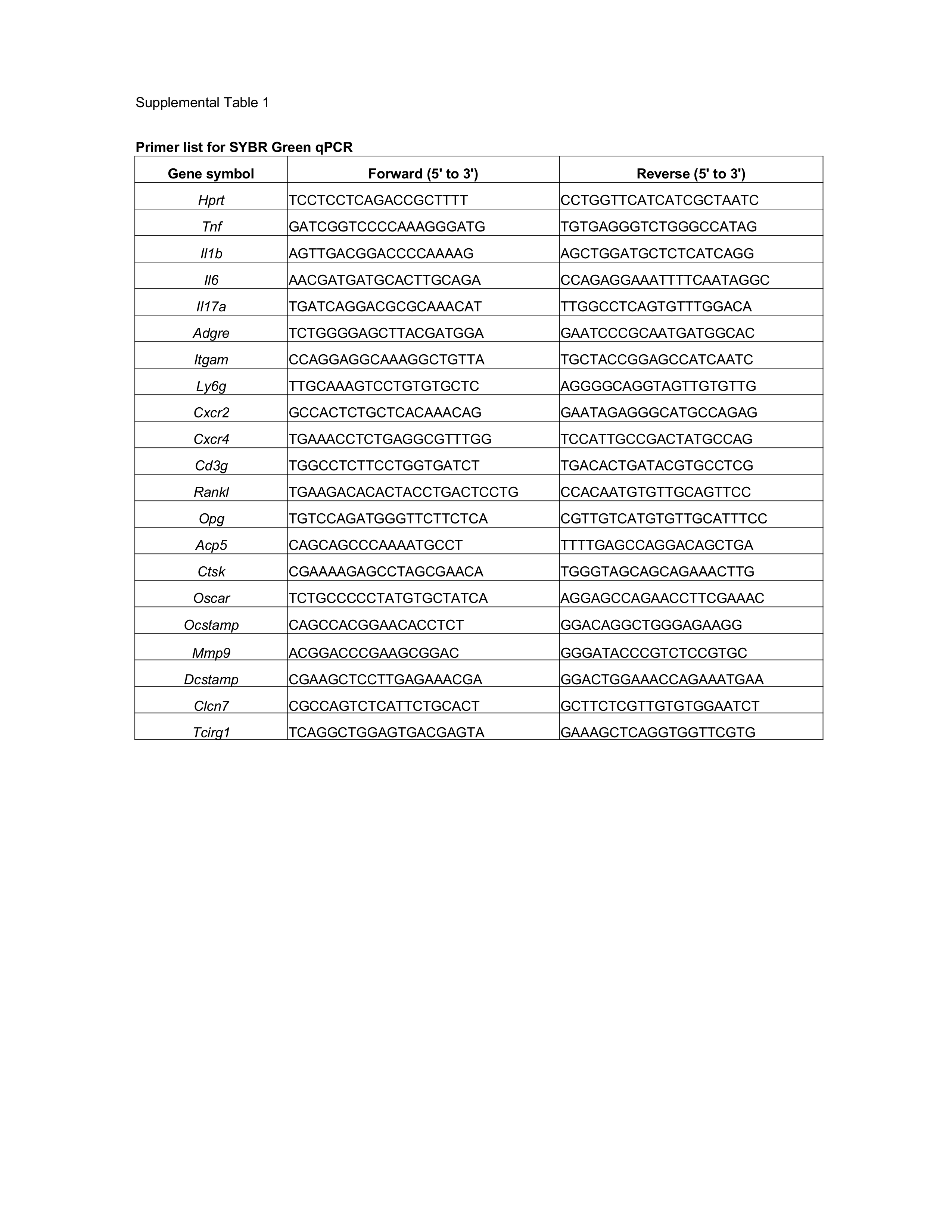

Supplement: Supplementary file 1 — Table S1. Primer list for SYBR Green qPCR [file JBM4-4-e10352-s001.tiff]

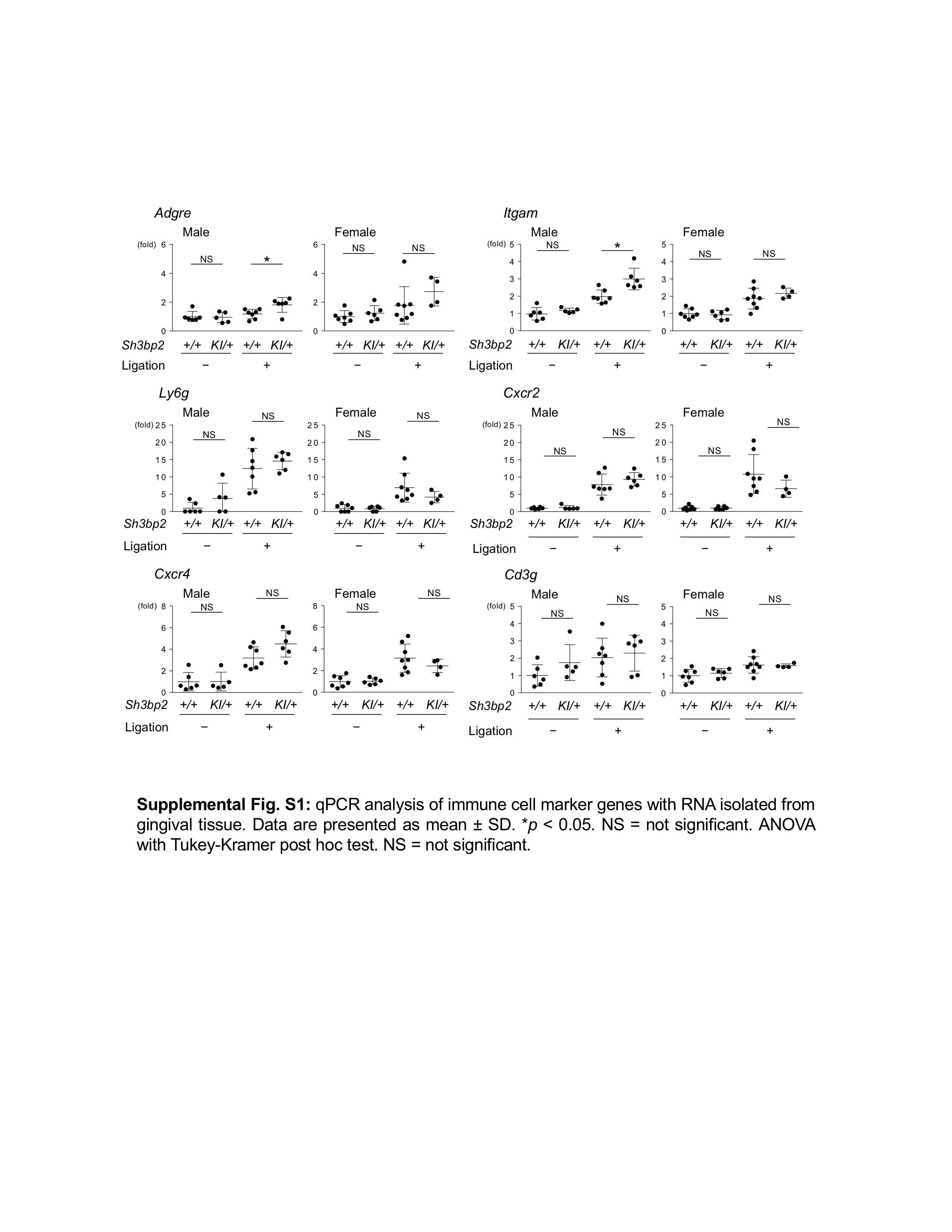

Supplement: Supplementary file 2 — Figure S1. qPCR analysis of immune cell marker genes with RNA isolated from gingival tissue. Data are presented as mean ± SD. *p < 0.05. NS = not significant. ANOVA with Tukey‐Kramer post hoc test. NS = not significant. [file JBM4-4-e10352-s002.tiff]

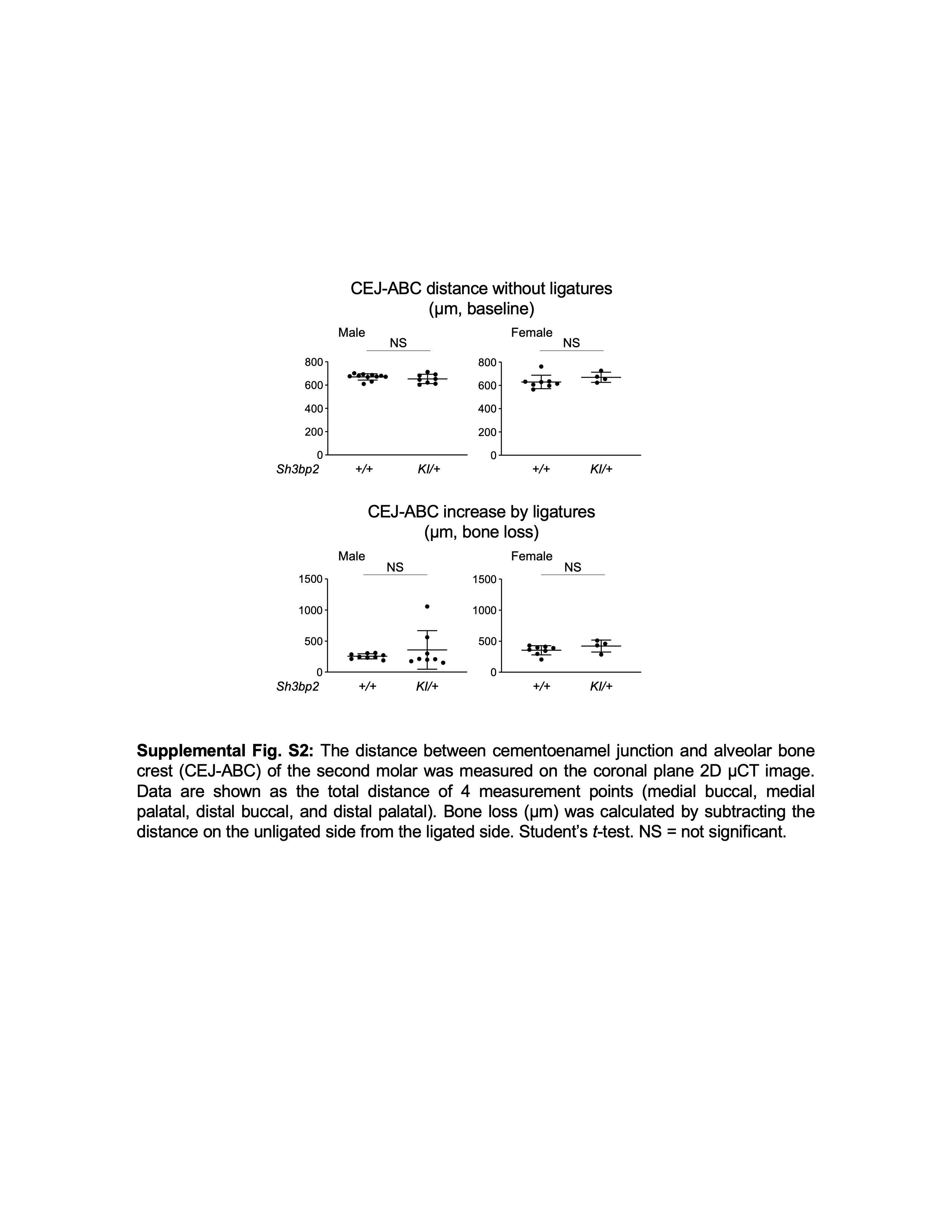

Supplement: Supplementary file 3 — Figure S2. The distance between cementoenamel junction and alveolar bone crest (CEJ‐ABC) of the second molar was measured on the coronal plane 2D μCT image. Data are shown as the total distance of 4 measurement points (medial buccal, medial palatal, distal buccal, and distal palatal). Bone loss (μm) was calculated by subtracting the distance on the unligated side from the ligated side. Student's t‐test. NS = not significant. [file JBM4-4-e10352-s003.tiff]

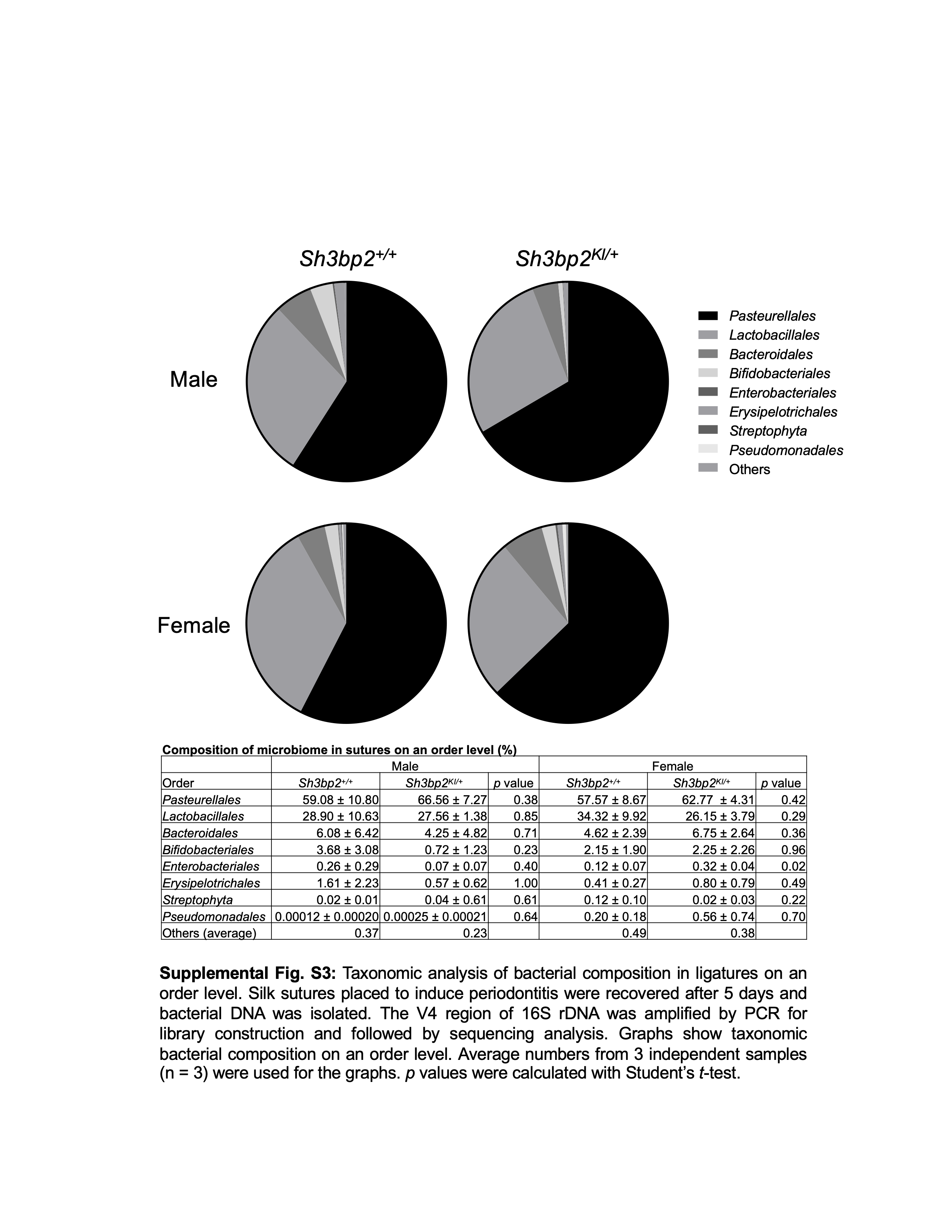

Supplement: Supplementary file 4 — Figure S3. Taxonomic analysis of bacterial composition in ligatures on an order level. Silk sutures placed to induce periodontitis were recovered after 5 days and bacterial DNA was isolated. The V4 region of 16S rDNA was amplified by PCR for library construction and followed by sequencing analysis. Graphs show taxonomic bacterial composition on an order level. Average numbers from 3 independent samples (n = 3) were used for the graphs. p values were calculated with Student's t‐test. [file JBM4-4-e10352-s004.tiff]

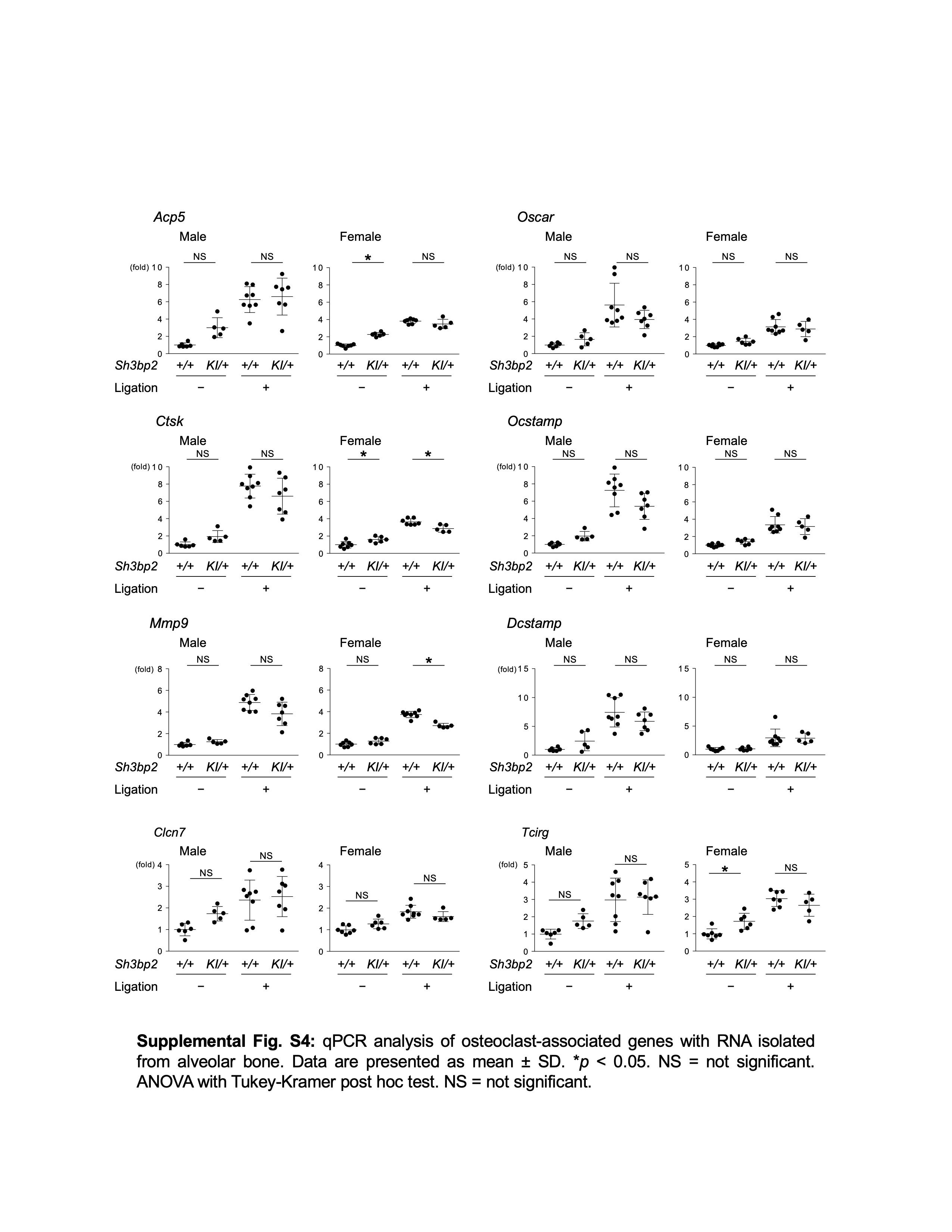

Supplement: Supplementary file 5 — Figure S4. qPCR analysis of osteoclast‐associated genes with RNA isolated from alveolar bone. Data are presented as mean ± SD. *p < 0.05. NS = not significant. ANOVA with Tukey‐Kramer post hoc test. NS = not significant. [file JBM4-4-e10352-s005.tiff]

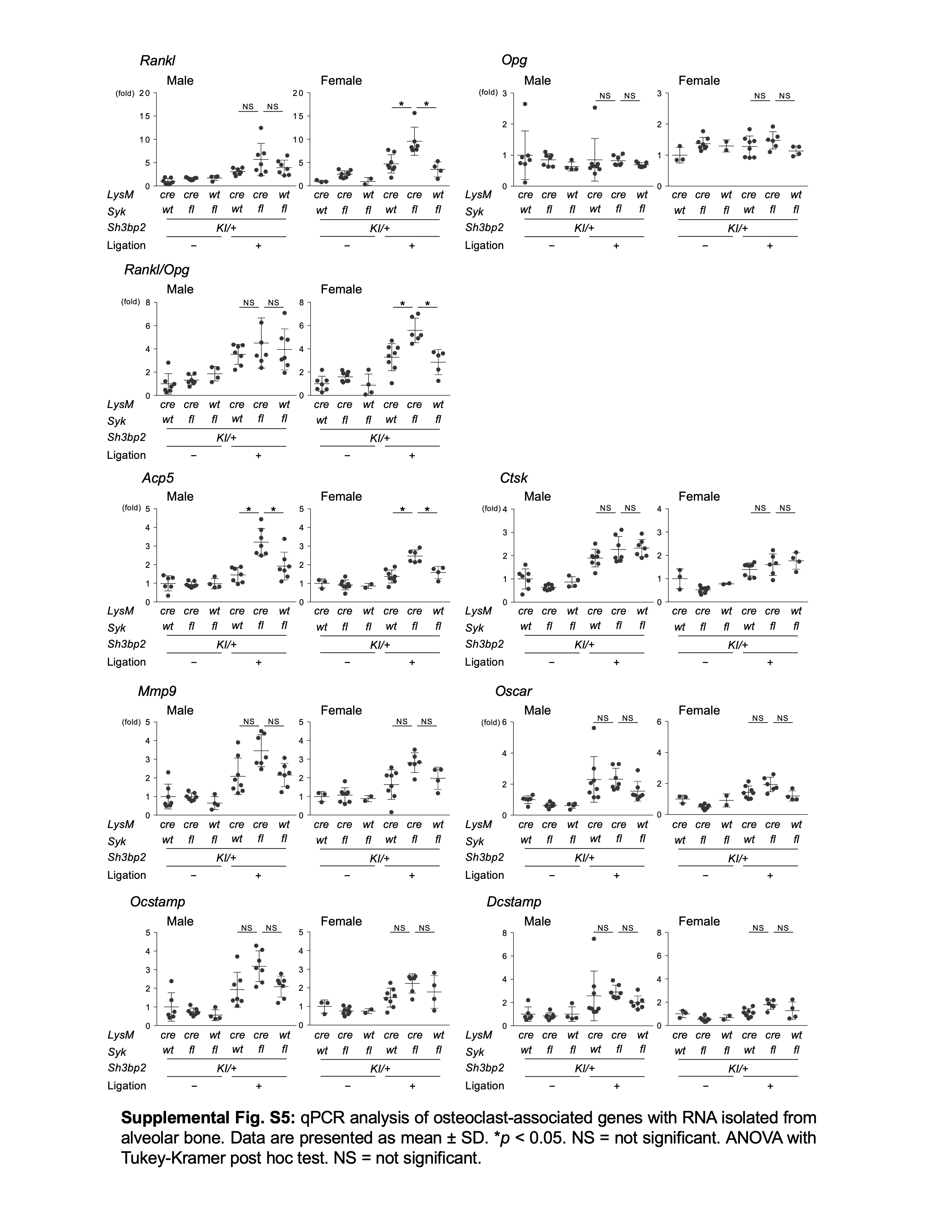

Supplement: Supplementary file 6 — Figure S5. qPCR analysis of osteoclast‐associated genes with RNA isolated from alveolar bone. Data are presented as mean ± SD. *p < 0.05. NS = not significant. ANOVA with Tukey‐Kramer post hoc test. NS = not significant. [file JBM4-4-e10352-s006.tiff]

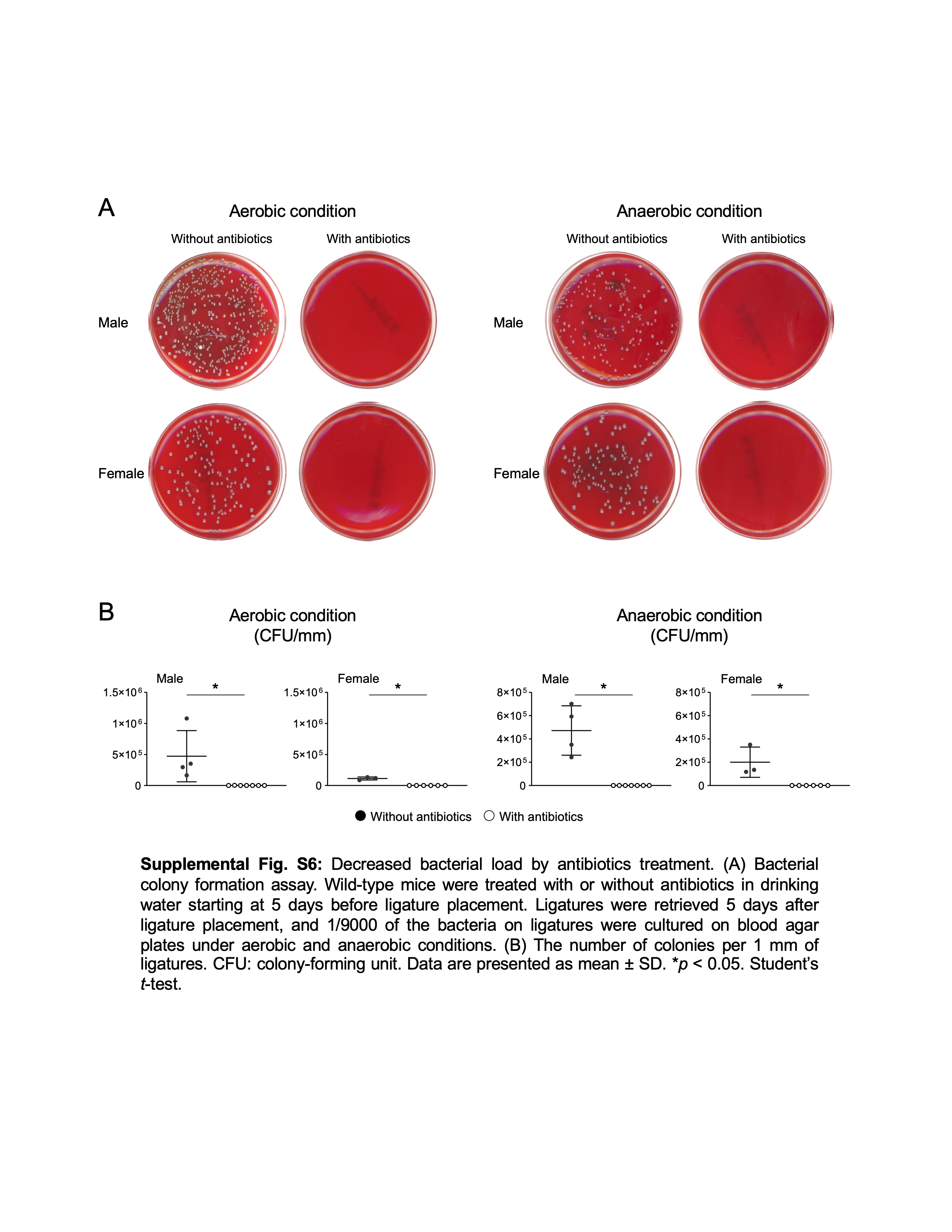

Supplement: Supplementary file 7 — Figure S6. Decreased bacterial load by antibiotics treatment. (A) Bacterial colony formation assay. Wild‐type mice were treated with or without antibiotics in drinking water starting at 5 days before ligature placement, and 1/9000 of the bacteria on ligatures were cultured on blood agar plates under aerobic and anaerobic conditions. (B) The number of colonies per 1 mm of ligatures. CFU: colony‐forming unit. Data are presented as mean ± SD. *p < 0.05. Student's t‐test. [file JBM4-4-e10352-s007.tiff]

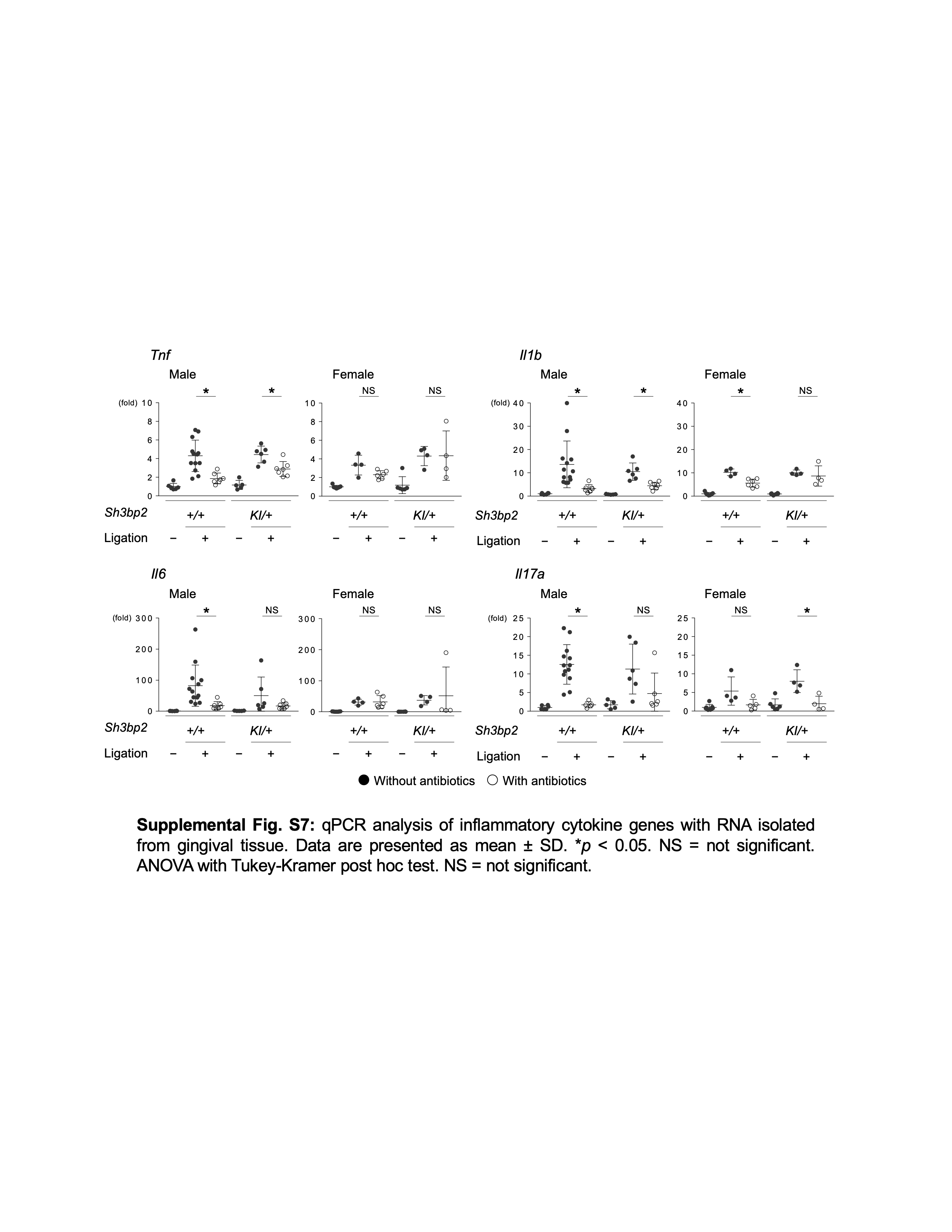

Supplement: Supplementary file 8 — Figure S7. qPCR analysis of inflammatory cytokine genes with RNA isolated from gingival tissue. Data are presented as mean ± SD. *p < 0.05. NS = not significant. ANOVA with Tukey‐Kramer post hoc test. NS = not significant. [file JBM4-4-e10352-s008.tiff]

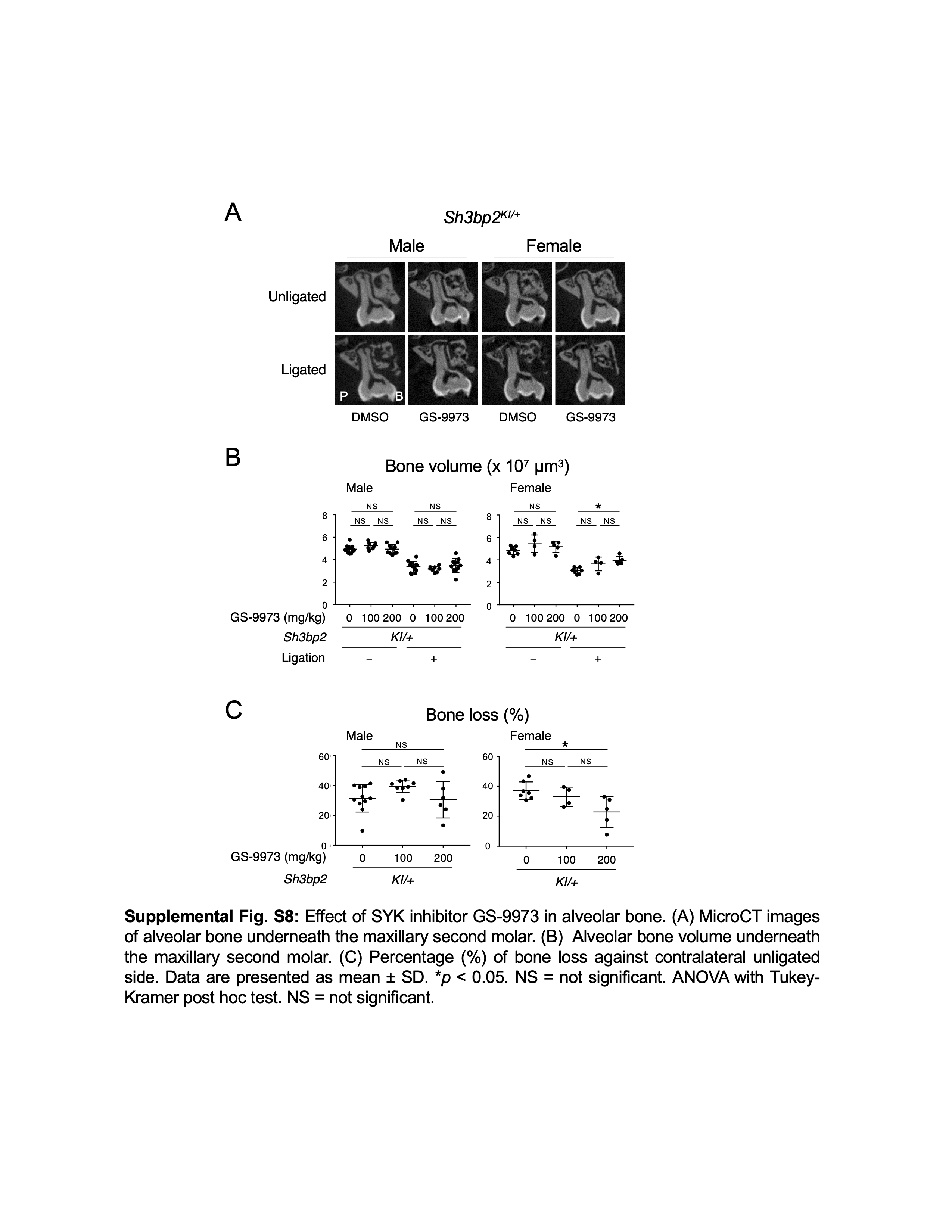

Supplement: Supplementary file 9 — Figure S8. Effect of SYK inhibitor GS‐9973 in alveolar bone. (A) MicroCT images of alveolar bone underneath the maxillary second molar. (B) Alveolar bone volume underneath the maxillary second molar. (C) Percentage (%) of bone loss against contralateral unligated side. Data are presented as mean ± SD. *p < 0.05. NS = not significant. ANOVA with Tukey‐Kramer post hoc test. NS = not significant. [file JBM4-4-e10352-s009.tiff]

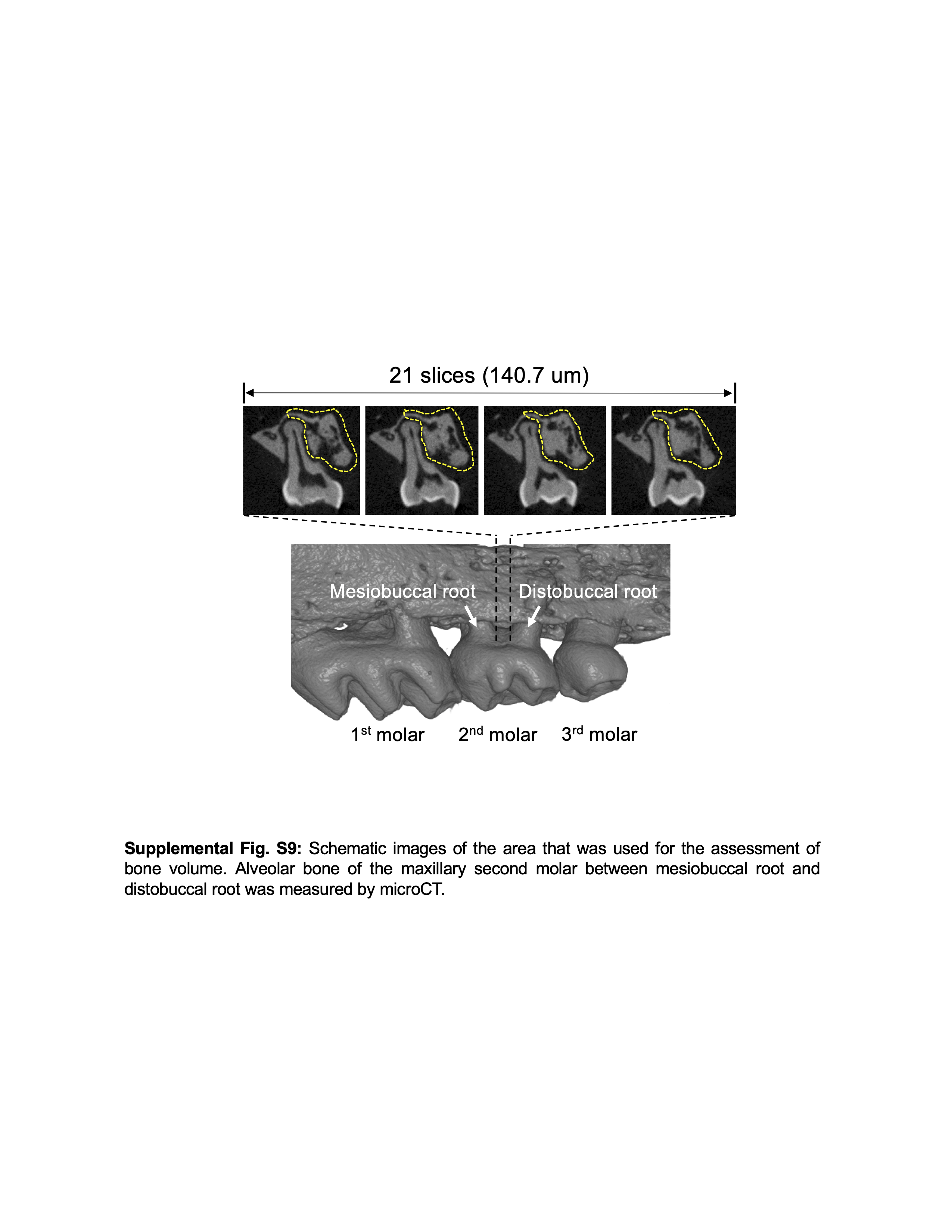

Supplement: Supplementary file 10 — Figure S9. Schematic images of the area that was used for the assessment of bone volume. Alveolar bone of the maxillary second molar between mesiobuccal root and distobuccal root was measured by microCT. [file JBM4-4-e10352-s010.tiff]
